# Identical Fe–N_4_ Sites with Different Reactivity: Elucidating the Effect of Support Curvature

**DOI:** 10.1021/acsami.4c19913

**Published:** 2025-01-29

**Authors:** Zdeněk Jakub, Jakub Planer, Dominik Hrůza, Azin Shahsavar, Jiří Pavelec, Jan Čechal

**Affiliations:** † CEITEC−Central European Institute of Technology, Brno University of Technology, Purkyňova 123, Brno 61200, Czech Republic; ‡ Institute of Applied Physics, TU Wien, Wiedner Hauptst. 8-10/E134, Wien 1040, Austria; § Institute of Physical Engineering, Faculty of Mechanical Engineering, Brno University of Technology, Technická 2896/2, Brno 61200, Czech Republic

**Keywords:** single atom catalysis, 2D metal−organic frameworks, scanning tunneling microscopy, density functional theory, adsorption, Fe−N4 site

## Abstract

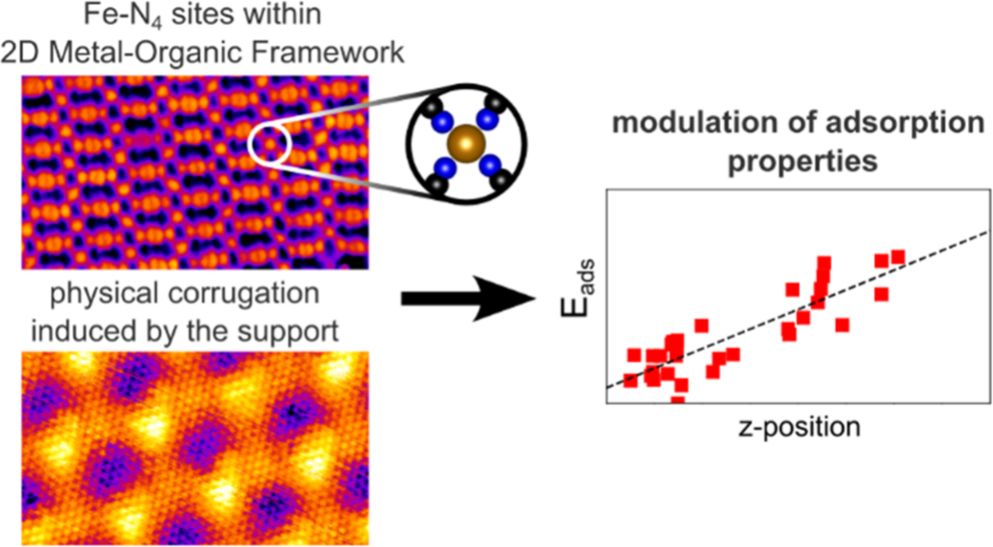

Detailed atomic-scale understanding is a crucial prerequisite for rational design of next-generation single-atom catalysts (SACs). However, the sub-ångström precision needed for systematic studies is challenging to achieve on common SACs. Here, we present a two-dimensional (2D) metal–organic system featuring Fe–N_4_ single-atom sites, where the metal–organic structure is modulated by 0.4 Å corrugation of an inert graphene/Ir(111) support. Using scanning tunneling microscopy and density functional theory, we show that the support corrugation significantly affects the reactivity of the system, as the sites above the support “valleys” bind TCNQ (tetracyanoquinodimethane) significantly stronger than the sites above the “hills”. The experimental temperature stability of TCNQ varies by more than 60 °C, while computations indicate more than 0.3 eV variation of TCNQ adsorption energy across the Fe–N_4_ sites placed atop different regions of the corrugated graphene unit cell. The origin of this effect is steric hindrance, which plays a role whenever large molecules interact with neighboring single-atom catalyst sites or when multiple reactants coadsorb on such sites. Our work demonstrates that such effects can be quantitatively studied using model SAC systems supported on chemically inert and physically corrugated supports.

## Introduction

Single-Atom Catalysis (SAC) promises to dramatically reduce the need for precious metals and make emerging green technologies economically feasible.^1−3^ Some of the most promising SACs of today are based on nitrogen-doped carbon materials, in which the metal atoms bind to the dopant sites.^4−7^ One of the commonly reported active sites features a transition metal atom bound to four nitrogen atoms, commonly referred to as metal-N_4_.^8−10^ The experimental data of N-doped carbon SACs show truly impressive results, often surpassing the state-of-the-art catalysts in both activity and selectivity.^11,12^

The atomic-scale identification of the reaction mechanisms typically relies on density functional theory (DFT) computations, which use simplified models featuring metal-N_*x*_ sites. The activity of a catalyst is intimately linked to the adsorption strength of the individual reactants and reaction intermediates; this relationship is qualitatively described by the well-known Sabatier principle.^13^ Over the past decades, a great effort has been invested in predicting reaction rates from selected computed “descriptor” values.^13−16^ However, this approach is potentially problematic for N-doped carbon SACs, as recent literature clearly indicates that many parameters can significantly affect the computed adsorption properties of the metal-N_*x*_ sites: the presence of local defects or additional atoms near the metal-N_*x*_ sites,^17−20^ local strain,^16,21^ or material above or below the computed monolayer.^22^ All these effects are undoubtedly present in the working SAC systems, but systematic experimental studies are challenging because the working catalysts are typically not characterized with resolution high enough to unambiguously identify and assess these fine and local structural details. To a certain degree, the adsorption properties of SAC sites can be correlated to their electronic structure (e.g., the position of the d-band or individual d-orbitals), but an equally important role is played by the physical structure of the active site and its structural evolution upon reactant adsorption.^23^ In this work, we use a detailed surface science approach to quantify the effects of sub-ångström structural distortions on the reactivity of single-atom catalyst sites. We utilize an atomically defined two-dimensional (2D) metal–organic framework (MOF) placed atop a physically corrugated support, and we show that already an <0.4 Å height corrugation of the 2D MOF structure significantly changes the adsorption properties of the system.

The employed 2D MOF consists of Fe atoms coordinated to tetracyanoquinodimethane (TCNQ) molecules, exposing well-defined Fe–N_4_ sites. The underlying graphene/iridium(111) support is chemically inert but physically corrugated by 0.4 Å due to the lattice mismatch between the graphene and Ir(111). By a combined scanning tunneling microscopy (STM) and density functional theory (DFT) study, we show that TCNQ probe molecules adsorb on the 2D MOF preferentially above the low-lying “valley” areas of the underlying graphene. Specifically, the Fe–N_4_ sites residing close to the “hill” areas of the corrugated graphene were only found to be occupied at temperatures lower than 90 °C, while the sites near the “valley” areas could keep the TCNQ molecules stable up to temperatures close to 150 °C. DFT computations confirm that even a small 0.4 Å corrugation of the chemically inert support causes an 0.3 eV difference in TCNQ adsorption energy atop Fe-TCNQ. DFT modeling allows us to track this difference down to steric hindrance, which destabilizes the large TCNQ molecule bound to the Fe–N_4_ sites residing near the protruding “hill” regions of the graphene. Overall, our work demonstrates a way to quantitatively study the effects of steric hindrance, which play an important role whenever large molecules interact with neighboring “single-atom” catalyst sites or when multiple reactants coadsorb on such sites.

## Results and Discussion

**Figure 1:**
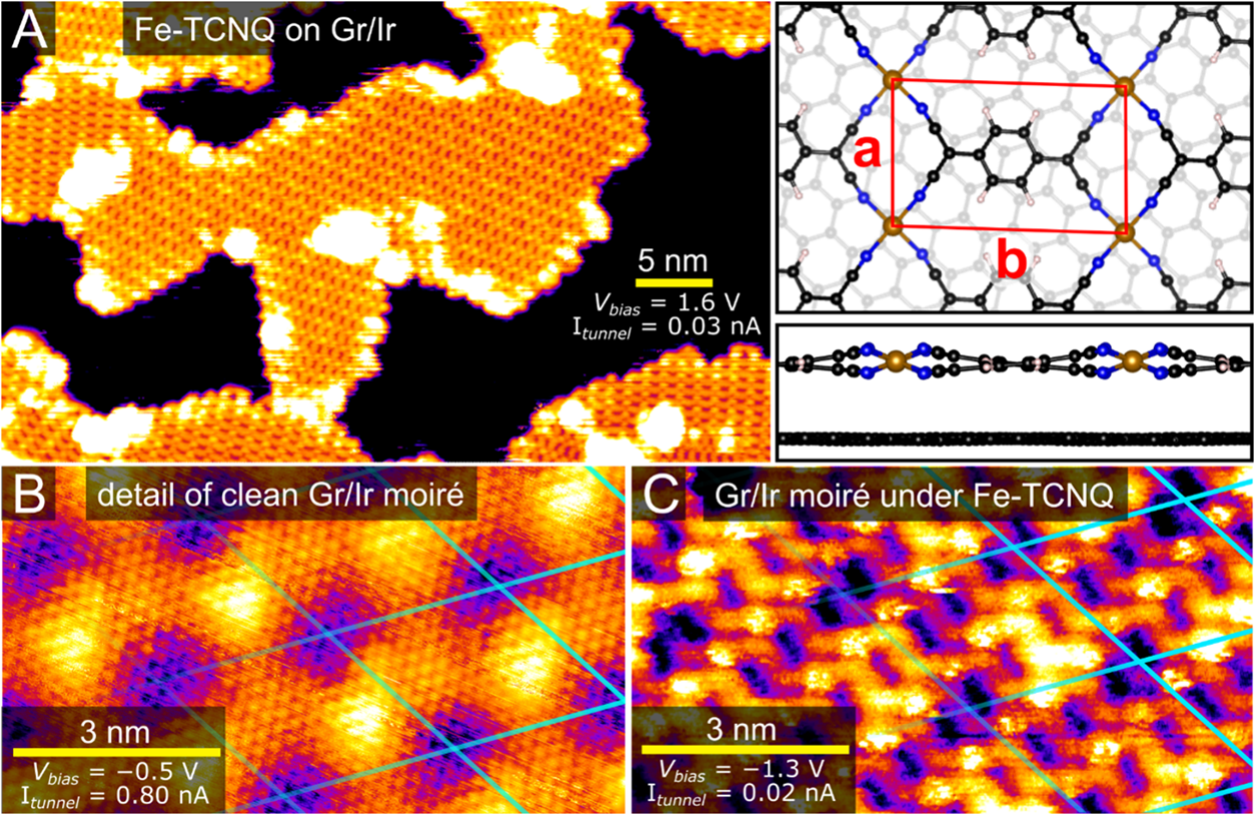
STM and DFT characterization of the Fe-TCNQ system supported on graphene/Ir(111). (A) An STM image of Fe-TCNQ on Gr/Ir. The corresponding DFT model is shown in the right panel (see ref (25) for details). (B) Detailed STM image of the pristine graphene/Ir(111) support, resolving both the graphene lattice and the long-range moiré pattern originating from lattice mismatch with the Ir(111) support (highlighted by the cyan grid). (C) The periodicity of the Gr/Ir moiré pattern is also visible in STM images of the Fe-TCNQ, as highlighted by the cyan grid. The image was filtered to remove high-frequency noise.

Figure 1A shows an STM image of the Fe-TCNQ 2D MOF synthesized atop graphene/Ir(111) (further abbreviated as Gr/Ir), using a protocol described in the Methods section and ref (24). The Fe-TCNQ structure is present in several rotational domains with typical dimensions of tens of nanometers. It was shown previously that this 2D MOF system is thermally and chemically stable, as it survives annealing temperatures above 450 °C as well as exposure to ambient conditions. Detailed STM images show that the Fe atoms are bound to four cyano groups and are thus similar to the square-planar Fe–N_4_ sites commonly present on N-doped carbon SACs.^24,25^ Previous work has also demonstrated that the Fe–N_4_ site geometry within Fe-TCNQ on graphene is not perfectly square-planar but resembles a quasi-tetrahedral motif, as shown in the DFT model in the right panel of Figure 1A.^25^ This nonplanarity is most likely caused by the preference of the high-spin Fe^2+^ center to reside in tetrahedral geometry, which can be accommodated due to the structural flexibility of the 2D MOF and its weak interaction with the graphene support. Alternate tilting of TCNQ linkers results in local (2 × 1) periodicity visible at specific scanning conditions (not visible in Figure 1A; details provided in Supporting Note 1 and in ref (25)). The spacing of the Fe-sites is regular with the nearest-neighbor distances of ≈7 and 11 Å in the directions labeled as *a* and *b* in the inset of Figure 1A.

Figure 1B shows a detailed STM image of the Gr/Ir support, where both the atomic structure of the graphene (lattice constant 2.46 Å) and the long-range moiré periodicity (lattice constant 25.3 Å, cyan grid) arising from the lattice mismatch between the graphene and the Ir(111) support is resolved. It is well established that within the Gr/Ir moiré unit cell, the graphene is corrugated by 0.4–0.5 Å, while the local work function varies by 0.1 eV.^26,27^ Finally, Figure 1C shows a detailed STM image of the Fe-TCNQ/Gr/Ir system, revealing that the Gr/Ir moiré periodicity can also be observed on the Fe-TCNQ 2D MOF supported on Gr/Ir. Specifically, a subtle contrast modulation is observed on the TCNQ molecules, with the same periodicity as the underlying moiré. This is highlighted in Figure 1C by the cyan grid; at each grid point, the TCNQ molecule is slightly darker. However, it is important to note that the Gr/Ir moiré was not observed to play a significant role in the Fe-TCNQ synthesis. Also, previous work has shown that TCNQ does not preferentially adsorb in any specific area on the clean Gr/Ir moiré.^28^

**Figure 2:**
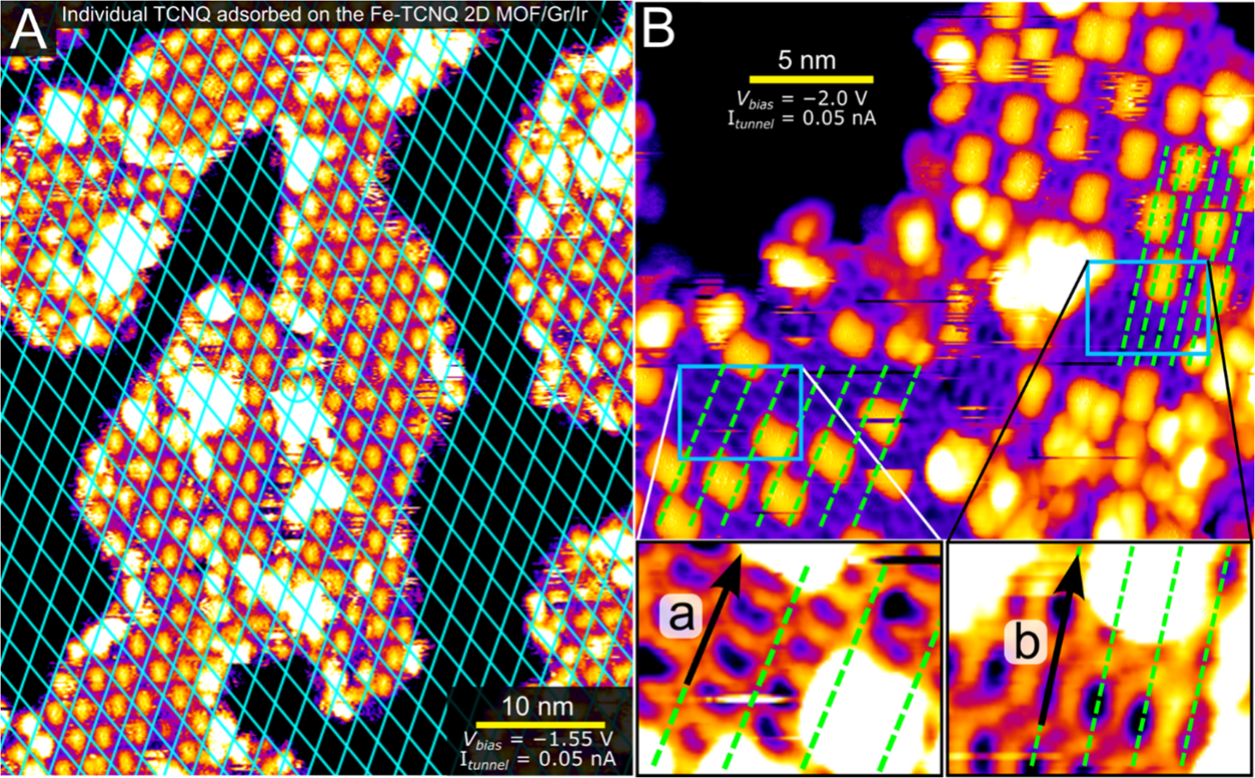
Individual TCNQ molecules adsorbed on Fe-TCNQ/graphene/Ir(111). The sample was prepared by deposition of additional TCNQ molecules on Fe-TCNQ/Gr/Ir and postannealing to 140 °C (A) An STM image overlaid with a grid with the periodicity of graphene/Ir moiré; the positions of individual TCNQ molecules adsorbed on the 2D MOF follow the grid. (B) A detailed STM image reveals the lateral position of adsorbed TCNQ with respect to the Fe-TCNQ. The adsorbed TCNQ molecules are located directly above the linker molecules within the Fe-TCNQ structure, as highlighted by the green lines connecting the Fe atoms in **a** and **b** directions. The adsorbed TCNQ molecules are always located in the middle between the dashed lines. The zoomed-in insets are contrast-enhanced for better visibility of the Fe-TCNQ structure.

Having a well-defined 2D MOF system featuring a dense array of Fe–N_4_ sites, we proceed to adsorption studies. We chose TCNQ as a probe molecule because it strongly chemically interacts with the Fe–N_4_ sites, allowing us to conduct a detailed study at or above room temperature. Figure 2A shows an STM image after deposition of >1 monolayer (ML) of TCNQ atop 2D MOF Fe-TCNQ/Gr/Ir and postannealing to 140 °C. This postannealing treatment leads to the desorption of weakly bound TCNQ molecules. The TCNQ molecules remaining on the surface are imaged as bright, elongated protrusions that appear regularly spaced atop Fe-TCNQ with the average distance between the individual monomers similar to the spacing of the Gr/Ir moiré. The image in Figure 2A highlights the spacing of the preferred adsorption sites by a cyan grid: The majority (>85%) of well-resolved TCNQ molecules reside in the grid windows, only rarely crossing the grid lines. Within experimental uncertainty, the cyan grid has lattice parameters identical to the Gr/Ir moiré, which strongly suggests that the spacing of TCNQ monomers is caused by the underlying moiré pattern. Additional analysis supporting this conclusion is provided in Supporting Note 2. In a series of STM images taken subsequently in the same area, we observe mobility of the adsorbed TCNQ species. This observation indicates that diffusion readily takes place at room temperature (at least in the vicinity of the STM tip), but adsorption sites above specific regions of the Gr/Ir moiré are preferred.

Figure 2B shows a high-resolution STM image of a sample prepared following the same procedure. Here, the Fe-TCNQ structure can be resolved in the areas free of adsorbed TCNQ, as shown in the zoomed-in, contrast-enhanced images in blue rectangles. This allows us to identify that the adsorbed TCNQ is preferentially located directly above a TCNQ molecule within the Fe-TCNQ structure, as highlighted by the dashed green lines connecting the Fe atoms in **a** direction (bottom left region of the image) and **b** direction (middle-right region of the image) of the Fe-TCNQ unit cell. The adsorbed TCNQ molecules are always located midway between two lines. This STM data set thus conclusively shows that the adsorbed TCNQ molecules are located above the linker molecules within the Fe-TCNQ structure, and they follow the rotational orientation of the underlying linker molecules.

**Figure 3:**
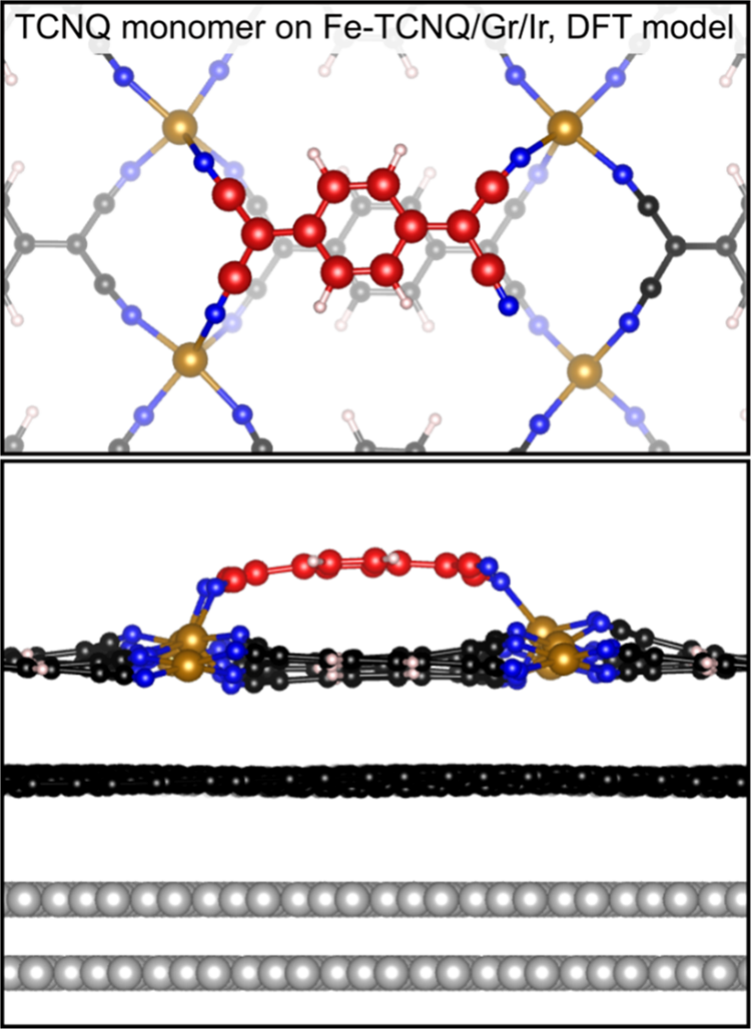
DFT model of a single TCNQ molecule adsorbed on Fe-TCNQ/graphene/Ir. The C atoms of the adsorbed TCNQ molecule are highlighted in red color. The Ir atoms are gray, C atoms (of graphene and Fe-TCNQ) are black, N atoms blue, Fe atoms brown, and H atoms white.

The geometry of TCNQ residing atop the Fe-TCNQ observed in Figure 2 is consistent with a DFT model shown in Figure 3 (the adsorbed TCNQ molecule is highlighted in red). We find that the most stable configuration of adsorbed TCNQ is bound to the 2D MOF by three −CN-Fe bonds of equivalent bond length (2.15 ± 0.03) Å; the computed TCNQ adsorption energy is −2.36 eV. Importantly, our computational setup includes the underlying Ir(111) support, which allows us to model the effects of graphene corrugation on the 2D MOF adsorption properties. It needs to be noted, however, that our model has lower physical corrugation than the real experimental system due to approximations arising from the necessity to facilitate periodic boundary conditions (see Methods section and Supporting Note 5) and thus it underestimates the differences in site-specific TCNQ adsorption energies. Despite this limitation, we identify a significant >0.20 eV variation of TCNQ/Fe-TCNQ adsorption energy across the fully relaxed Gr/Ir unit cell. Then, as detailed below, the effect of larger support corrugations is estimated from fixed-graphene models, in which the increased physical corrugation leads to TCNQ adsorption energy differences of up to 0.5 eV at the experimentally relevant corrugation of 0.4–0.5 Å.

**Figure 4:**
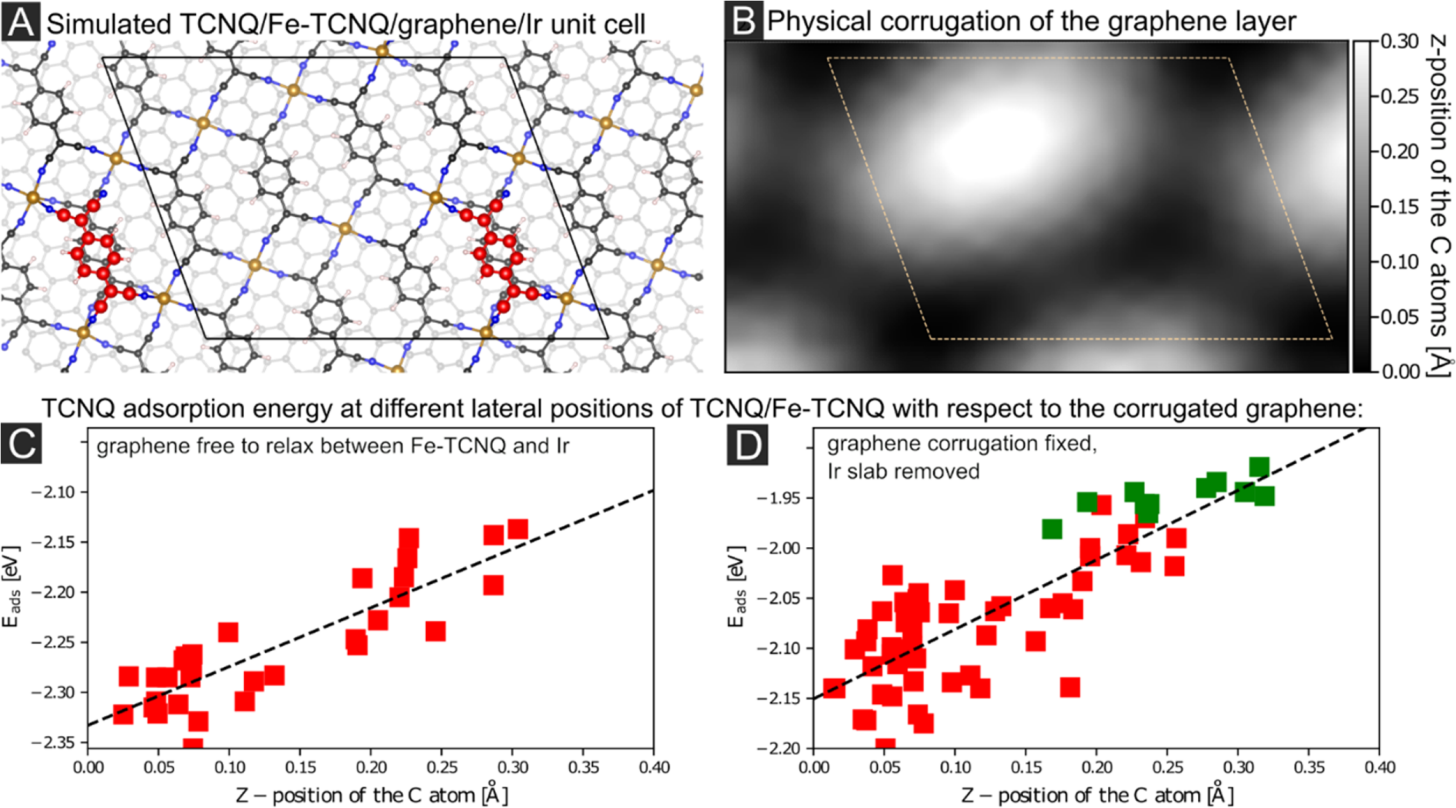
Computational models of TCNQ adsorption atop Fe-TCNQ/Gr/Ir. (A) The simulated unit cell contains eight Fe_1_(TCNQ)_1_ units atop a corrugated Gr/Ir moiré unit cell. The adsorbed TCNQ molecule is highlighted in red color. (B) The corrugation of the graphene layer; valley sites are dark and hill sites bright. The position of one of the calculated Fe-TCNQ unit cells is marked by a dashed parallelogram. (C) The dependence of TCNQ adsorption energy on the *z*-position of the underlying graphene obtained from fully relaxed DFT models. The linear fit of data points is given as a dashed line. (D) The dependence of TCNQ adsorption energy on the *z*-position of the underlying graphene when the Ir slab was removed and graphene corrugation fixed. The green points represent positions in which the most stable adsorption configuration featured only two – CN-Fe bonds. The trend given by a dashed line is qualitatively the same as in panel (C).

Figure 4A shows the simulated Fe-TCNQ/Gr/Ir unit cell with an adsorbed TCNQ molecule highlighted in red color (Ir slab not shown for clarity). The 0.3 Å corrugation of the graphene layer is plotted in grayscale in panel 4B. To study the effects of the relative position of TCNQ/Fe-TCNQ with respect to the Gr/Ir moiré, we calculated the TCNQ adsorption energy on all possible positions within the unit cell shown in Figure 4A; and then performed the calculations for different lateral positions of the Fe-TCNQ layer with respect to Gr/Ir moiré. Overall, more than 176 models were evaluated by DFT to safely identify the trends (details of the model generation are described in Supporting Note 6). Figure 4C shows the dependence of the TCNQ/Fe-TCNQ adsorption energy on the z-position of the underlying C atoms of the graphene support. The trend clearly shows that the most stable TCNQ/Fe-TCNQ sites are located in the low-lying (“valley”) areas of the Gr/Ir moiré.

To determine whether the changes in TCNQ adsorption energies are due to the physical or electronic corrugation of the Gr/Ir moiré, we conducted a set of computations in which the physical corrugation of the graphene was fixed, and the Ir slab was removed. Thus, the physical corrugation remains, but the electronic corrugation arising from the weak hybridization and charge transfer between the C 2*p*_*z*_ and Ir 5*d*_*z*^2^_ orbitals in the “valley” regions^29^ is absent. Figure 4D shows that the trend of TCNQ/Fe-TCNQ adsorption energies is the same as in the previous data set. Also, no significant differences are observed in the electronic structure of the individual TCNQ/Fe-TCNQ/Gr models (see Supporting Note 7). Thus, we conclude that it is primarily the physical corrugation of the underlying graphene that affects the adsorption properties of the supported 2D MOF, and already a 0.3 Å corrugation causes the adsorption energy of TCNQ to vary by more than 0.2 eV.

**Figure 5:**
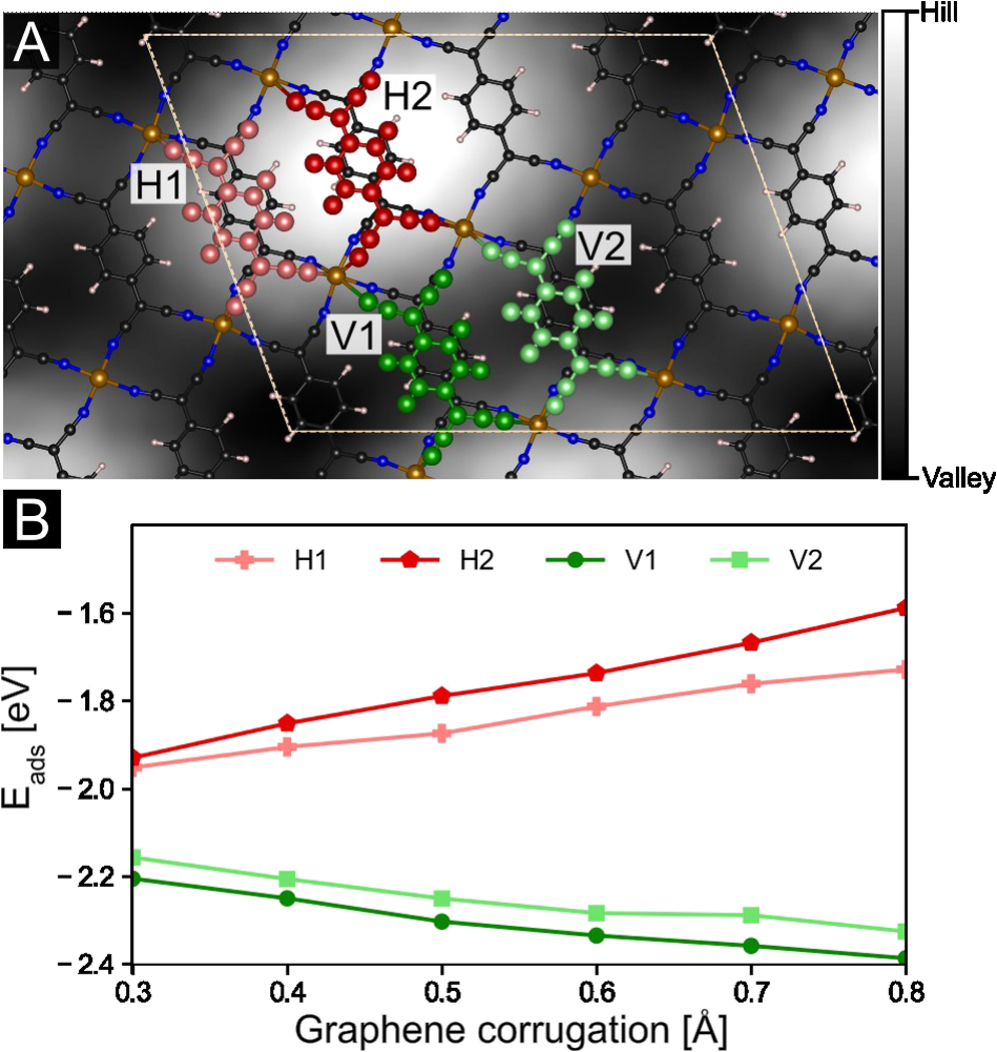
Variation of TCNQ adsorption energies with increasing graphene corrugation for four representative models of the TCNQ/Fe-TCNQ layer adsorbed on the graphene sheet. (A) Illustration of two TCNQ monomer models positioned above the “hill” of the underlying graphene (labeled H1 and H2, highlighted by red color) and two TCNQ monomers positioned above the “valleys” (labeled V1 and V2, highlighted by green color). The lateral position of the FeTCNQ layer is the same in these models. (B) Calculated adsorption energies of the TCNQ monomer in the four representative models as the function of the graphene corrugation. The increasing support corrugation stabilizes the TCNQ monomers above the “valleys”, and simultaneously destabilizes the TCNQ monomers above the “hills”.

The real experimental system has a corrugation higher than our fully relaxed model (0.4–0.5 Å vs 0.3 Å); thus, the differences in adsorption properties are expected to be even higher. To quantify this claim, we relaxed representative models of the TCNQ/Fe-TCNQ above the “valley” and “hill” areas of fixed graphene sheets with the height corrugation increasing stepwise from 0.3 Å to 0.8 Å. This data set, presented in Figure 5, indicates that the increasing support corrugation further stabilizes the TCNQ monomers adsorbed above the “valley” regions and simultaneously destabilizes the TCNQ monomers above the “hill” regions. When the support corrugation is comparable to the real experimental system (0.4–0.5 Å), the computed TCNQ adsorption energy differences are 0.3–0.5 eV, i.e., significantly higher than in the model with 0.3 Å corrugation.

**Figure 6:**
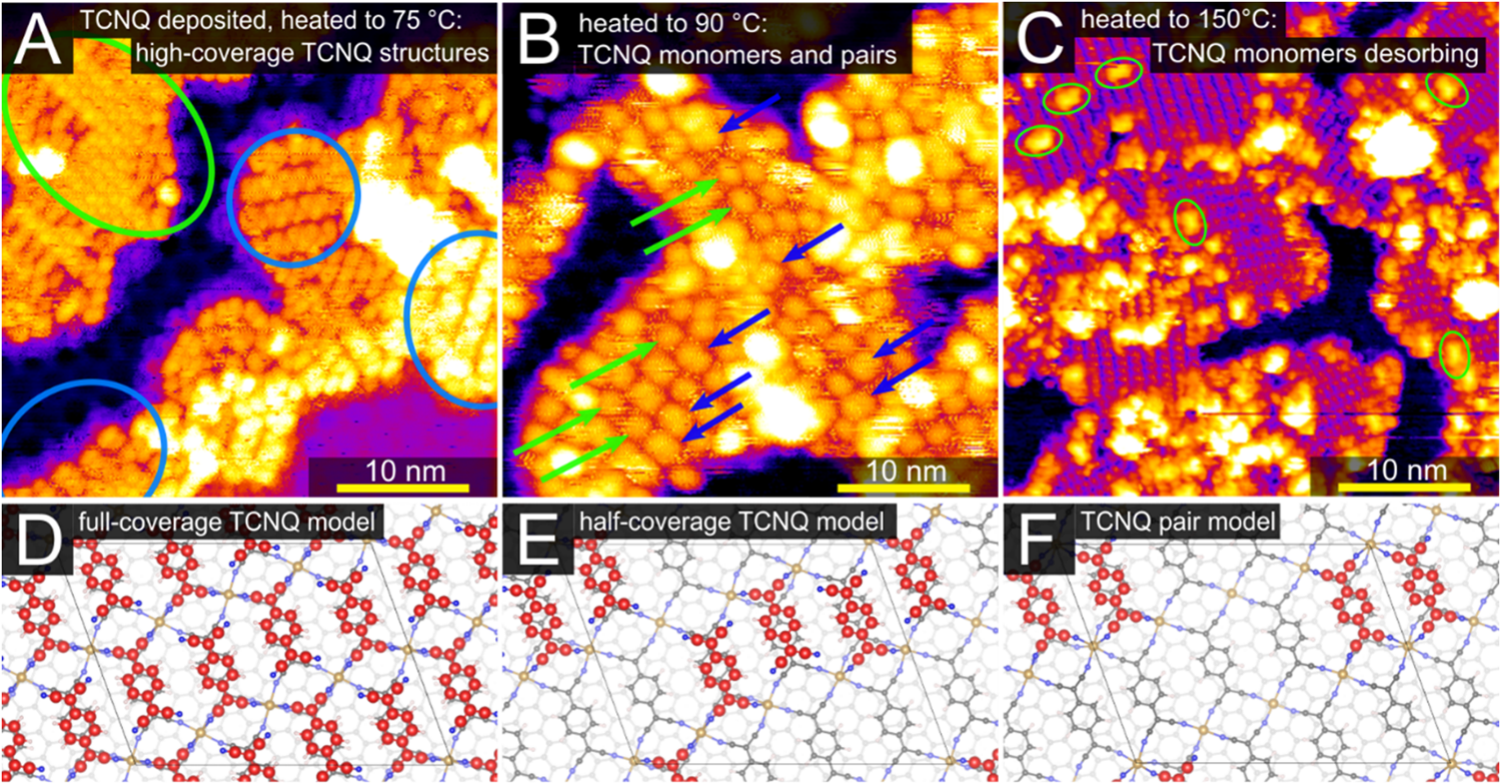
STM and DFT analysis of the structures with varying TCNQ/Fe-TCNQ coverage. (A) Upon TCNQ deposition and heating to 75 °C, STM images show areas fully covered with TCNQ (green oval, a corresponding DFT model shown in panel (D)) and areas with half-monolayer coverage. A phase with a zigzag appearance is highlighted by blue ovals; a corresponding DFT model is shown in panel (E). (B) Upon heating to 90 °C, the higher-coverage structures are desorbed, and isolated features are observed. These are interpreted as TCNQ monomers (green arrows) and pairs (blue arrows; the corresponding model is shown in panel (F)). (C) Upon heating to 150 °C, only a few TCNQ monomers are observed at the pristine Fe-TCNQ far from domain boundaries (green ovals). (D– F) DFT-optimized models of the higher-coverage TCNQ phases. The C atoms in adsorbed TCNQ are highlighted by red color. The scanning parameters of STM imaging are (A) *V* = −1.75 V, *I* = 0.05 nA, (B) *V* = −2.00 V, *I* = 0.05 nA, (C) *V* = −2.00 V, *I* = 0.05 nA.

We conducted further experiments with higher TCNQ coverage to find the limits of TCNQ stability in both the “valley” and “hill” regions of the Fe-TCNQ/Gr/Ir system. We find that a monolayer coverage of TCNQ molecules on Fe-TCNQ (i.e., one TCNQ above every linker in the 2D MOF structure) is only observed in patches after TCNQ deposition and postannealing below 75 °C (green oval in Figure 6A, additional data in Supporting Note 3); Figure 6D shows a plausible DFT-optimized model of this phase. We also identify several ordered phases of 0.5 monolayer TCNQ coverage, which are dominant upon postannealing to 75 °C (blue ovals in Figure 6A). Here, the sites above the “hill” regions are still occupied. One of these phases has a characteristic zigzag appearance (blue ovals in Figure 6A), which we interpret as neighboring TCNQ molecules in 2-fold coordination, as shown in the DFT model in Figure 6E.

Most of these ordered TCNQ structures are desorbed upon heating to 90 °C. The corresponding STM image presented in Figure 6B shows individual bright features spaced with the periodicity of the Gr/Ir moiré, similar to those observed in Figure 2. However, a significant fraction of these bright protrusions is larger than the previously observed TCNQ monomers. We interpret these as pairs of TCNQ molecules (blue arrows); these features are significantly wider than single TCNQ (highlighted by green arrows for comparison). These TCNQ dimers are relatively stable, as some of them are still clearly observed even upon heating to 140 °C (shown in Supporting Note 4). In our DFT computations (model shown in Figure 6F), the TCNQ pairs are energetically almost identical to two isolated monomers. In agreement with experimental observations, the DFT calculations also show a strong preference for TCNQ pairs to reside in the “valley” region compared to the “hill” (>0.17 eV per molecule).

Finally, Figure 6C shows an STM image after heating to 150 °C, where the remaining TCNQ monomers reside primarily at domain boundaries of the 2D MOF, but a few are still found on pristine Fe-TCNQ (green ovals in Figure 6C). We note that the low surface density of TCNQ monomers on pristine Fe-TCNQ is tricky to assess from room-temperature STM data due to the (possibly tip-induced) mobility of the TCNQ atop defect-free Fe-TCNQ. Regardless of this complication, we can safely conclude that the TCNQ molecules desorb from the ordered phases already upon heating to 90 °C, but at least a small number of TCNQ monomers remain above the “valley” regions up until 150 °C.

Overall, our work addresses the adsorption properties of single-atom catalyst sites and elucidates how strongly these are affected by small structural distortions. Our experimental and computational results clearly demonstrate that TCNQ molecules adsorbed at the 2D MOF sites above the “valley” regions of the underlying Gr/Ir moiré are bound significantly stronger than the ones adsorbed above the “hills”. This effect originates from a complex structural relaxation of the system upon TCNQ adsorption. This adsorbate-induced relaxation involves all the atoms within the Fe-TCNQ layer, and as such, it cannot be correlated to any single parameter like coordination number, bond length, or amount of transferred charge (see Supporting Notes 7 and 8). Nevertheless, it can be intuitively rationalized by steric hindrance: The adsorption of TCNQ involves coordination to multiple Fe–N_4_ sites, which lifts the Fe atoms upward and pushes the linker molecule toward the support. This is evident in DFT computations in which a free-standing planar graphene sheet is free to relax under the TCNQ/Fe-TCNQ (shown in Figure S13). Upon relaxation of this model, a ≈ 0.6 Å deep depression forms in the graphene layer directly underneath the TCNQ monomer, and this relaxation significantly stabilizes the system. This further indicates that the configuration of a TCNQ/Fe-TCNQ directly above a support depression is energetically favorable. In contrast, when the monomer is located above a different region in the underlying support, the system relaxes into a slightly different structure, and the sum of all the small changes in the atom positions within the simulated system leads to an overall significant increase in energy, i.e., decrease in stability. The overall energy difference arises both from the long-range dispersion forces between the graphene and TCNQ/Fe-TCNQ, and the slightly differing ligand field within the distorted TCNQ/Fe-TCNQ system (see Supporting Note 8).

Eventually, the most compelling evidence of the effect is provided by extensive DFT modeling presented in Figure 4, which shows a clear trend in the TCNQ monomer stability with the varying z-position of the underlying carbon atoms in the graphene support. Furthermore, this approach also provides confirmation that the effect is primarily structural and not electronic–the same trend was identified in models of corrugated graphene without the underlying Ir support, where the local work function of graphene is not modulated by the orbital overlap with the Ir support. This perfectly agrees with the fact that the features in the electronic structure of TCNQ/Fe-TCNQ are almost identical regardless of the TCNQ location with respect to the graphene (as summarized in Supporting Note 7). Based on these data sets, we can safely conclude that the electronic corrugation of the graphene/Ir does not play a significant role in this case, and the observed phenomenon is mainly due to steric effects.

The effects of steric hindrance take place in all applied systems, but their significance always depends on the exact system of interest: generally, whenever there are multiple chemical bonds formed (either due to multidentate adsorption of one molecule or due to multiple monodentate reactants coadsorbing on the same site), then we’d expect the steric hindrance to play an important role, to the point where even a tiny sub-ångström distortion can significantly affect the adsorption energy, as observed in this work. On the other hand, small monodentate adsorbates will likely be unsusceptible to such effect or would need a much higher support curvature to be affected. To qualitatively test this claim, we have carried out a computational study of ammonia (NH_3_) adsorption atop corrugated Fe-TCNQ/Gr, and we observed no correlation between the ammonia adsorption energy and the z-position of the underlying C atoms of the graphene support (data summarized in Supporting Note 9). This illustrates the fact that the surprisingly strong effect of support curvature in the sub-ångström range is restricted to large molecules in specific bonding configurations. Thus, we expect that this effect may play a significant role in catalytic reactions involving large molecules (for example, in organic synthesis) but will likely be less important for reactions involving small molecules (such as CO oxidation or ammonia synthesis).

Overall, the small sub-ångström structural details affecting adsorption properties of the individual SAC sites are nearly impossible to identify on working catalysts, but we have shown that surface science methodology enables an elegant way to quantitatively model these effects using 2D MOFs supported on chemically inert yet physically corrugated supports.

## Conclusions

We have studied the interaction of TCNQ molecules with the Fe–N_4_ “single-atom catalyst” sites embedded within an Fe-TCNQ 2D MOF supported on Gr/Ir support. Even though the Fe–N_4_ sites appear identical at first sight and are better defined than in most real systems, we experimentally observed significant differences in adsorption energies of TCNQ residing on the Fe-TCNQ above the “valley” and “hill” areas of the Gr/Ir moiré. The Fe–N_4_ sites residing close to the “hill” areas of the moiré were only found to be occupied at temperatures lower than 90 °C, while the sites near the “valley” areas could keep the TCNQ molecules stable up to temperatures close to 150 °C. Our DFT computations confirm that even a small 0.3 Å corrugation of the inert support causes a >0.20 eV difference in TCNQ adsorption energy atop Fe-TCNQ. This adsorption energy difference linearly increases with physical corrugation, reaching 0.4–0.5 eV at a corrugation of 0.5 Å. DFT modeling further indicates that the stability of adsorbed TCNQ monomers is primarily influenced by the physical corrugation of the graphene support, rather than electronic modulation. We rationalize our observations by steric hindrance, and we conclude that similar effects will take place whenever large molecules interact with neighboring sites in “single-atom” catalysts or when multiple reactants coadsorb on the same site. Overall, we have shown that the synthesis of 2D systems atop chemically inert yet physically corrugated supports presents a viable strategy to study such effects with unprecedented precision. This paves the way toward systematic studies required for further development of single-atom catalysts.

## Methods

Experiments were carried out in an ultrahigh vacuum system consisting of multiple chambers interconnected by a central transfer line, separated by gate valves. The base pressure of all the chambers used in this study is below 5 × 10^–10^ mbar. The Ir(111) single crystals (supplied by MaTecK and SPL) were cleaned by cycles of Ar^+^ sputtering (1.5 keV, 10 min) and flashing up to 1300 °C, followed by annealing to 1150 °C (10 min). When graphene was present on the sample prior to cleaning, the first annealing cycle took place in O_2_ background (1000 °C, *p*_O2_ = 1 × 10^–6^ mbar). The temperature was measured by a LumaSense IMPAC IGA 140 pyrometer with the emissivity set to 0.1.

Graphene on Ir(111) was grown by adsorbing saturation coverage of ethylene at room temperature, followed by pumping out the ethylene background and ramping the temperature up to 1250 °C in UHV. Then, the sample was re-exposed to ethylene at this temperature (1 × 10^–6^ mbar, 5 min). This protocol combines temperature-programmed growth (TPG) with chemical vapor deposition (CVD),^30,31^ and consistently leads to a full monolayer coverage of high-quality graphene/Ir(111).

For the Fe-TCNQ synthesis, TCNQ was thermally evaporated from a quartz crucible heated to 115 °C (MBE Komponenten OEZ), and iron was evaporated from an effusion cell (MBE Komponenten HTEZ). The evaporation rate of metals was checked by a water-cooled quartz crystal microbalance. The temperature during Fe-TCNQ synthesis was calibrated by a special sample holder with a K-Type thermocouple attached close to the crystal surface. The Fe-TCNQ synthesis protocol involved saturation of the substrate by TCNQ at a temperature close to a TCNQ desorption temperature, followed by the codeposition of Fe and TCNQ at the same temperature. The TCNQ desorption temperature was determined by XPS measurements and was found to be ≈80 °C on graphene/Ir(111). After the codeposition, the samples were annealed to 420 °C, unless stated otherwise in the text. This annealing step leads to improved long-range order of the Fe-TCNQ 2D MOF and also leads to the desorption of TCNQ molecules adsorbed atop the 2D MOF. A detailed analysis of graphene-supported Fe-TCNQ systems is provided in refs (24) and (25). The additional TCNQ was deposited atop Fe-TCNQ using the same evaporator settings as during the MOF synthesis, the postannealing temperatures are given in the text.

Scanning tunneling microscopy images were recorded at room temperature in the constant current mode using a commercial system Aarhus 150 (SPECS) equipped with a Kolibri sensor using a tungsten tip. Distortion in the STM images was corrected to fit the known dimensions of the graphene/Ir moiré unit cell. Where possible, nonlinear image distortion was corrected as described in ref (32).

We performed density functional theory (DFT) calculations employing the Vienna Ab Initio Simulation package^33^ using the projector-augmented wave (PAW) method^34^ for treating core electrons. The exchange-correlation interaction was described by the nonlocal van der Waals corrected optPBE-vdW functional.^35^ Furthermore, we added a Hubbard-like coulomb repulsion parameter of 4 eV in Dudarev’s formulation^36^ to appropriately describe strongly localized Fe 3d orbitals. In contrast to our previous work,^25^ the energy cutoff for a plane-wave basis set was reduced from 520 to 460 eV, and the Brillouin zone was sampled with a single Γ point instead of 2 × 2 × 1 Γ-centered grid. These parameters ensure much faster calculations while giving almost identical formation energies of Fe-TCNQ layer on a graphene substrate (differences are below 0.008 eV per Fe_1_(TCNQ)_1_ unit). Geometries of studied structures were optimized until residual forces acting on atoms were smaller than 0.02 eV/Å.

For interface calculations, a corrugated graphene sheet with and without 2 layers of Ir(111) below was considered as a substrate, and a 15 Å thick vacuum slab was added to avoid interactions between periodically repeated replicas. The 2-layer thick Ir slab is sufficient to model the graphene corrugation; adding another 2 layers of Ir increases the graphene corrugation only by 0.03 Å. All the presented calculations account for dipole corrections to the potential, forces, and energy. To reduce the size of the resulting supercell, we modified the shape of the graphene moiré pattern while maintaining the orientations of the Gr/Ir and FeTCNQ/Gr interfaces consistent with experimental data; structural details are provided in Supporting Note 5. For calculations without the iridium support, the corrugation of the graphene sheet was first obtained by a full relaxation on an Ir substrate and then kept fixed. For estimation of the increasing graphene corrugation (shown in Figure 5), the *z*-coordinates of the carbon atoms were renormalized to result in the desired corrugation; the Fe-TCNQ structures with and without TCNQ were then relaxed on these fixed graphene sheets.

## Data Availability

The data underlying this study are available in the published article and its Supporting Information. The primary experimental data sets and used computational models are openly available in the Zenodo repository at https://doi.org/10.5281/zenodo.14753673.

## Supporting Information Available

Additional STM data showing the nonplanarity of Fe-TCNQ/gr/Ir; additional analysis of the TCNQ monomer spacing; larger-scale images of high-coverage TCNQ/Fe-TCNQ phases; the existence of TCNQ pairs after annealing to higher temperatures; details of the DFT computations and design of the TCNQ/Fe-TCNQ/Gr/Ir models; comparison of specific parameters within the TCNQ/Fe-TCNQ/Gr models with different stability; DFT computations of NH_3_ adsorption on Fe-TCNQ/Gr (PDF)
