# Supplementary material for: Identical Fe–N4 Sites with Different Reactivity: Elucidating the Effect of Support Curvature
Source: ACS Appl Mater Interfaces. 2025 Jan 29;17(6):10136–44. doi: 10.1021/acsami.4c19913 (PMC11826504; doi:10.1021/acsami.4c19913)
Supplement: Supplementary file 1 — am4c19913_si_001.pdf [file am4c19913_si_001.pdf]

Supporting Information

Identical Fe-N_4 Sites with Different Reactivity: Elucidating the Effect of Support Curvature

Zdeněk Jakub[1‡*], Jakub Planer[1‡*], Dominik Hrůza[1], Azin Shahsavar[1], Jiří Pavelec[2], Jan Čechal[1,3]

[1] CEITEC – Central European Institute of Technology, Brno University of Technology, Purkyňova 123, 61200 Brno, Czech Republic

[2] Institute of Applied Physics, TU Wien, Wiedner Hauptst. 8-10/E134, 1040 Wien, Austria

[3] Institute of Physical Engineering, Faculty of Mechanical Engineering, Brno University of Technology, Technická 2896/2, Brno 61200, Czech Republic

‡ These two authors contributed equally

*Corresponding authors:
e-mail: zdenek.jakub@ceitec.vutbr.cz, jakub.planer@ceitec.vutbr.cz

Table of Contents

Supporting Note 1:

The non-planar Fe-TCNQ structure on graphene/Ir measured by STM 2

Supporting Note 2:

Further analysis of the TCNQ monomer spacing 2

Supporting Note 3:

Additional STM data of the high-coverage TCNQ/Fe-TCNQ phases 4

Supporting Note 4:

Presence of adsorbed TCNQ pairs after annealing above 100 °C 6

Supporting Note 5:

Structural model for the FeTCNQ/Graphene/Iridium(111) interface 6

Supporting Note 6:

Details of the computed TCNQ monomer models atop Fe-TCNQ/Gr and Fe-TCNQ/Gr/Ir 8

Supporting Note 7:

Analysis of the electronic structure differences between the models 11

Supporting Note 8:

Additional analysis of the origin of the adsorption energy differences 13

Supporting Note 9:

Influence of the graphene corrugation on NH_3 adsorption atop Fe-TCNQ 14

Supporting Note 1:

The non-planar Fe-TCNQ structure on graphene/Ir measured by STM

Figure S1 shows STM images of Fe-TCNQ/Gr/Ir measured at negative sample bias values between −1.3 eV and −2.0 eV. In the left panel, one can locally see the "zig-zag" appearance, which is due to the ordered non-planar TCNQ linkers within the Fe-TCNQ structure, as described in detail in reference [1]. The "zig-zag" pattern has a 14 × 11 Å periodicity (2**a** × **b**) and is also locally observed in large-scale STM images, as shown in the right panel of Figure S1.

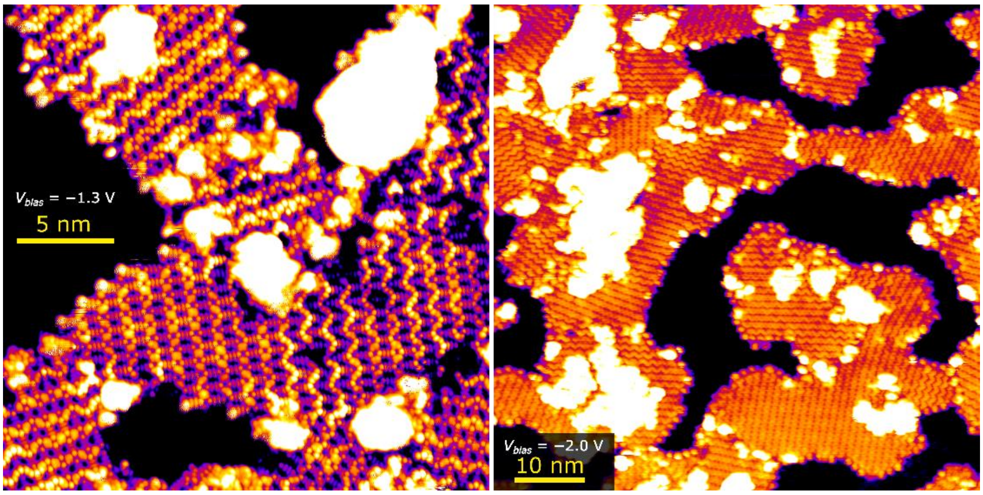

Figure S1: Room-temperature STM data showing the distinct "zig-zag" appearance of Fe-TCNQ/Gr/Ir.

Supporting Note 2:

Further analysis of the TCNQ monomer spacing

Figure S2 shows the same STM image as shown in Figure 2A in the main text but with the TCNQ monomers being highlighted by yellow and pink dots. The dot positions were identified using an automated Feature Finder script implemented in ImageJ.[2] The yellow dots indicate the TCNQ monomers are positioned within the grid windows. The pink dots were selected manually as the features crossing the grid lines. Overall, only ≈ 14 % (20 out of 145) of the features are crossing grid lines. Thus, more than 85% of the well-resolved monomers within this image reside within the grid windows, indicating a preference for some long-range ordering.

Additionally, we have taken the positions of the identified monomers and plotted them in a binary image where the monomer positions are single black points on a white background (see Figure S3A; for clarity, these single black points were highlighted by yellow dots). Then, we carried out a Fast Fourier Transformation of this binary image to identify any hints of spatial periodicity. The resulting FFT pattern is shown in Figure S3B, indicating a faint hexagonal pattern that can be overlaid with a grid corresponding to real-space periodicity of a = 2.56 nm, b = 2.45 nm, φ = 121°. Within the measurement uncertainty, these parameters are identical to the Gr/Ir moiré periodicity identified from the same image (Figure 3C, D, a = 2.56 nm, b = 2.54 nm, φ = 121°). We consider this unambiguous proof that the TCNQ/FeTCNQ monomer positions follow the periodicity of the underlying Gr/Ir moiré.

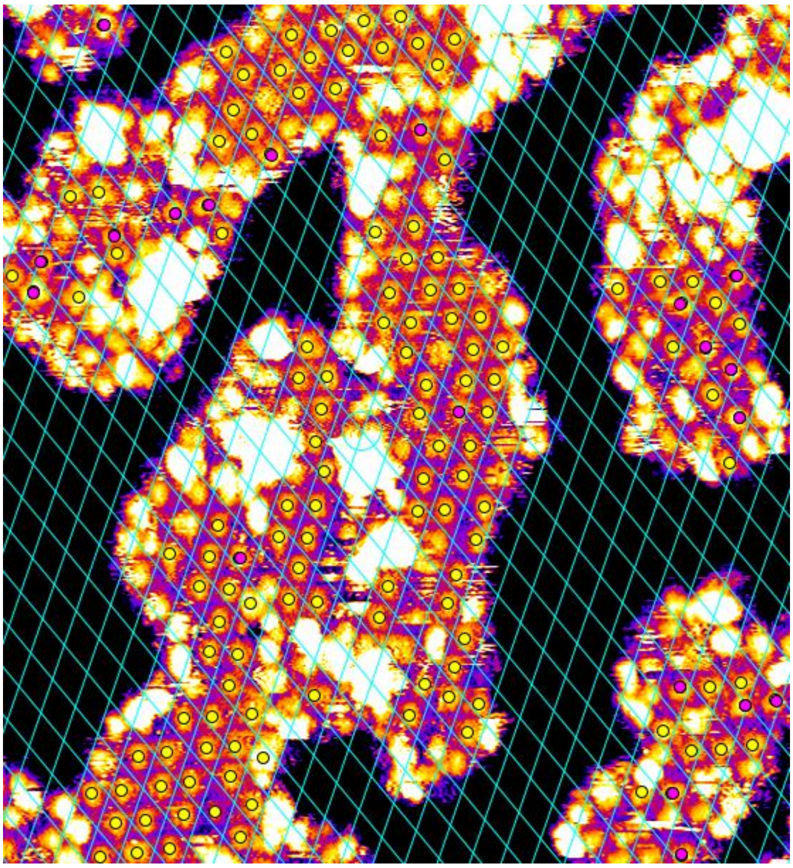

Figure S2: STM image of TCNQ monomers atop Fe-TCNQ/Gr/Ir, highlighting the positions of the individual TCNQ monomers. As described in the text, >85% of the monomers reside within the grid windows, hinting at some long-range order.

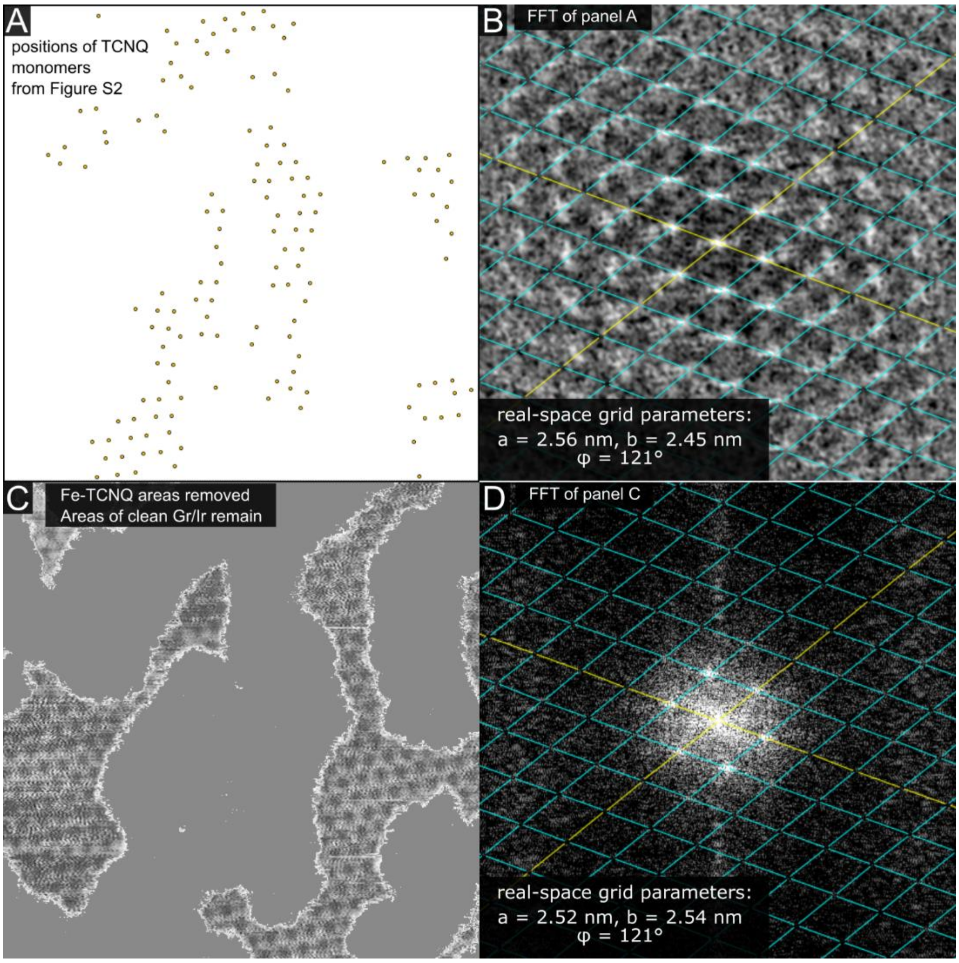

Figure S3: Additional analysis of the long-range ordering of TCNQ moiré positions atop Fe-TCNQ. (A) A binary image indicating the TCNQ monomer positions in the STM image shown in Figure S2. The TCNQ monomer positions are single black points on a white background (here, the single black points are highlighted by yellow dots for better visibility). (B) An FFT pattern of the binary image shown in panel A. A faint hexagonal pattern is observed, with the spatial periodicity identical (within uncertainty) to that of the Gr/Ir moiré. (C) An STM image from Figure S2, in which the areas of Fe-TCNQ were replaced by a constant gray background, and the areas of Gr/Ir were contrast-enhanced. (D) An FFT pattern of Figure S3C. A clear hexagonal pattern is found, originating from the Gr/Ir moiré periodicity.

Supporting Note 3:

Additional STM data of the high-coverage TCNQ/Fe-TCNQ phases

Figure S4 shows an STM image taken after Fe-TCNQ/Gr/Ir preparation, additional TCNQ deposition, and post-annealing to 65 °C, i.e., below the TCNQ desorption from Gr/Ir. One can see that the TCNQ molecules are also adsorbed on the areas of bare graphene. On the Fe-TCNQ 2D MOF, we primarily observe a structure with a periodicity similar to the full-coverage phase shown in Figure 6A in the main text, i.e., showing 2×1 periodicity with respect to the Fe positions (this structure is highlighted by green ovals). Additionally, we observe small patches of an alternative phase with a larger unit cell (blue ovals), which most likely correspond to the "zig-zag" phase with 0.5 ML of TCNQ coverage on the Fe-TCNQ 2D MOF (see Figure 6A and 6E in the main text).

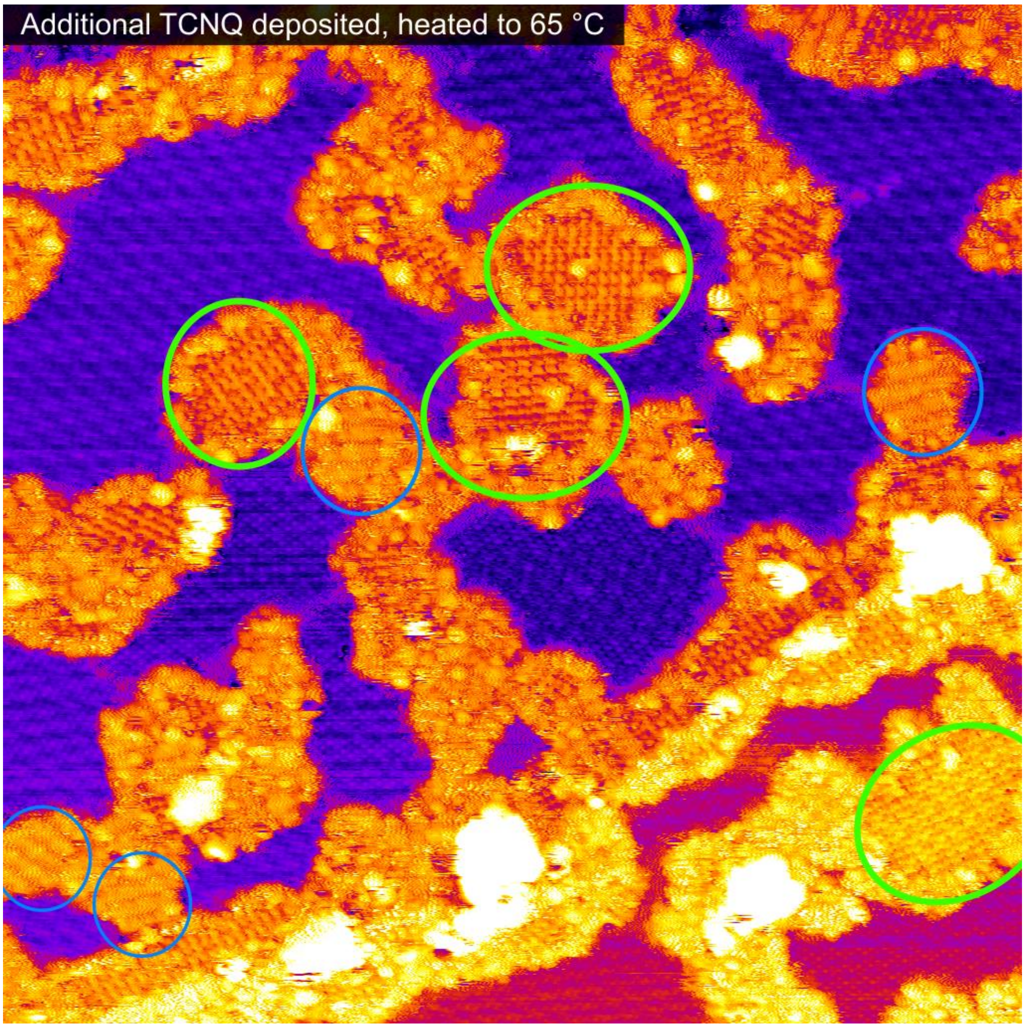

Figure S4: Room-temperature STM image of a Fe-TCNQ/Gr/Ir system after additional TCNQ deposition and post-annealing to 65 °C. The scanning area is 100×100 nm^2, the scanning parameters are V_{bias}=–1.3 V, I_{tunnel}=0.05 nA

Supporting Note 4:

Presence of adsorbed TCNQ pairs after annealing above 100 °C

Figure S5 shows STM images of Fe-TCNQ 2D MOF on Gr/Ir after deposition of additional TCNQ and post-annealing to 140 °C. This treatment results in the presence of TCNQ monomers atop the Fe-TCNQ 2D MOF, but a significant number of TCNQ pairs is still observed, as highlighted by the green circles.

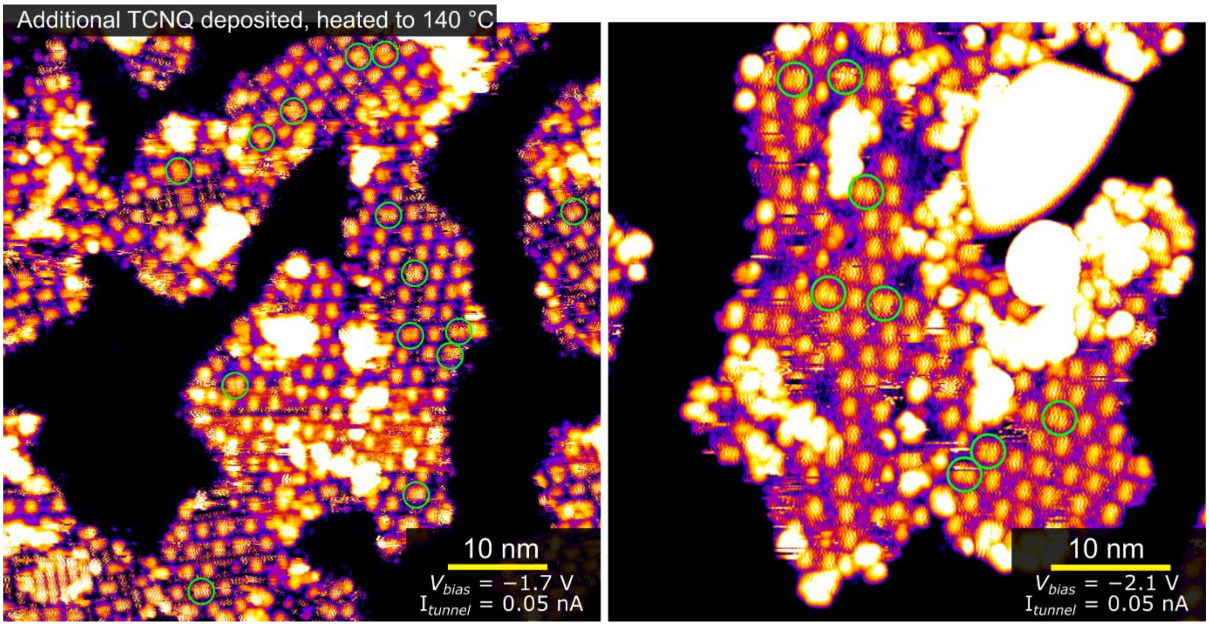

Figure S5: The presence of TCNQ pairs is still observed upon post-annealing to 140 °C, as highlighted by the green circles.

Supporting Note 5:

Structural model for the FeTCNQ/Graphene/Iridium(111) interface

The experimental Gr/Ir moiré supercell is known to be incommensurate, being formed by 10.32×10.32 graphene unit cells resting on 9.32×9.32 Ir(111) unit cells.[3] In DFT computations, this is commonly approximated as 10×10 Gr on 9×9 Ir(111), but this model does not offer a reasonable match with the FeTCNQ lattice. Thus, the inclusion of the Fe-TCNQ layer would lead to a large supercell that is unaffordable for calculations. Therefore, we approximated the shape and the size of the graphene moiré supercell while keeping orientations of FeTCNQ and graphene layers in line with the experiment using the unit cell described in Figure S6. Ir(111) and Fe-TCNQ layers were commensurated with the graphene sheet, causing small strains in both layers, as listed in Table ST1. The relaxed supercell with lateral dimensions of a = 30.3 Å, b = 22.6 Å, γ = 110 ° shows a graphene corrugation height of ≈ 0.3 Å; i.e., 0.1-0.2 Å lower than the experimental supercell. We modeled the iridium substrate with a 2-layered slab, as doubling the thickness to 4 layers increased the graphene corrugation only by 0.03 Å.

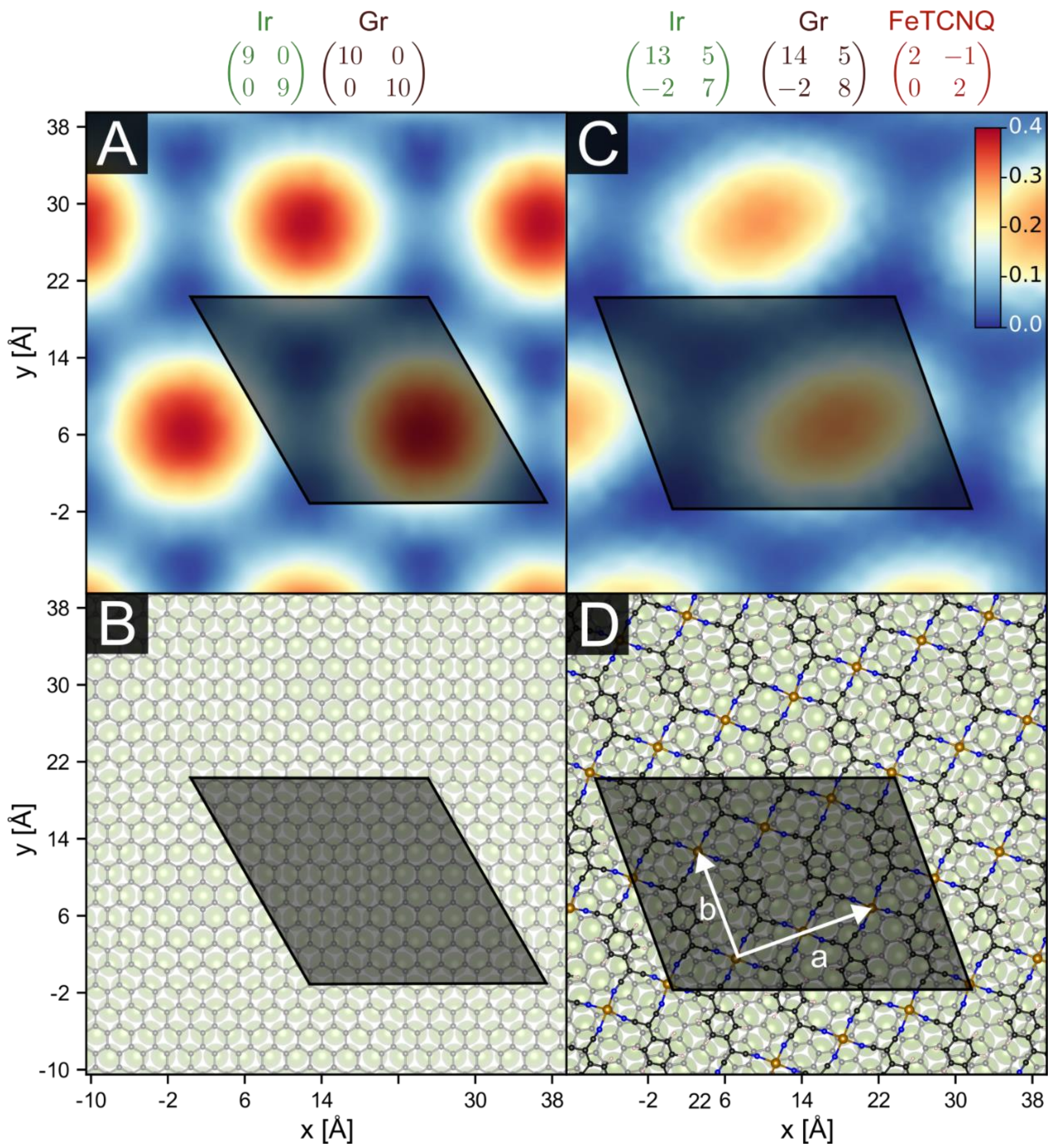

Figure S6: Relaxed geometries and epitaxy matrices for (A-B) experimental and (C-D) modified Gr-Moiré supercells employed in DFT calculations. Colormaps in panels A and C denote the z-position of C atoms in the graphene sheet.

Table ST1: Lengths of primitive vectors of graphene, Ir(111), and Fe-TCNQ layer observed experimentally and modified FeTCNQ/Gr/Ir(111) supercells. Negative and positive values in parenthesis denote compressive and tensile strains in commensurated layers, respectively. For details of free-standing and supported Fe-TCNQ structures, see reference [1].

	experimental graphene moiré			modified graphene moiré		
	Ir(111)	Graphene	FeTCNQ	Ir(111)	Graphene	FeTCNQ
a [Å]	2.71	2.44 (-0.9%)	-	2.76 (1.6%)	2.46	7.1 (1.4%)
b [Å]	2.71	2.44 (-0.9%)	-	2.67 (-1.5%)	2.46	11.3 (5.8%)
γ [°]	60.0	60.0	-	59.6 (0.7%)	60.0	88.4 (-1.8%)

Supporting Note 6:

Details of the computed TCNQ monomer models atop Fe-TCNQ/Gr and Fe-TCNQ/Gr/Ir

Overall, 128 models of TCNQ monomers on Fe-TCNQ/Gr were evaluated by a DFT optimization on a fixed graphene sheet to elucidate the adsorption energy trends, and 48 models were evaluated by DFT optimization of both the Fe-TCNQ and Gr on an Ir slab. The models were designed as follows: We placed a TCNQ molecule atop every one of the eight adsorption sites in the supercell shown in Figure 4A in the main text. On each of these sites, we tested four distinct adsorption geometries: a 3-fold coordinated TCNQ, a 2-fold coordinated TCNQ, and their "flipped" variants of the Fe-TCNQ; the term "flipped" is related to the orientation of the "zig-zag" pattern originating from the non-planarity of the Fe-TCNQ layer, as graphically shown in Figure S7. Then, we laterally shifted the TCNQ/Fe-TCNQ layer with respect to the corrugated graphene unit cell along the **b** direction, three times by ⅛ of the unit cell size, as marked with a black, green, and red arrow in Figure S8A. For each lateral shift, all eight adsorption sites, each with four possible configurations, were calculated. In these calculations, a fixed corrugated graphene sheet without the Ir(111) slab was employed as a substrate. The positions (centers of mass of the adsorbed TCNQ) and adsorption energies of the 64 3-fold coordinated TCNQ configurations are plotted in Figures S8B-C (the energies of the 2-fold configurations are plotted later in Figure S9). The dots and squares represent the centers of mass of the adsorbed TCNQ molecule; the plotted numbers are the energy differences (in meV) between the given adsorption configuration and the most stable configuration in the dataset. In most sites, the 3-fold coordination is preferred, but the 2-fold geometry is slightly more stable above the "hill" regions of the Gr/Ir moiré. This is shown in Figure S8B, where the squares indicate positions for which a more favorable 2-fold coordinated alternative exists. The same is marked in Figure S8C by green squares.

The calculations that include the Ir slab were done for 48 representative models of 3-fold and 2-fold coordinated TCNQ molecules, as depicted in Figure S6D-F. We found that the graphene relaxation stabilizes the representative 3-fold configurations by (190 ± 20) meV and the 2-fold ones by (110 ± 20) meV. Relaxing the graphene sheet thus leads to an overall increase of the calculated adsorption energies and stabilization of 3-fold TCNQ geometries with respect to 2-fold ones but does not change the identified trend.

Figure S9 compares the calculated adsorption energies between 2-fold and 3-fold coordinated TCNQ molecules using the fixed corrugated graphene (left panel) and Gr/Ir (right panel) as a substrate. Both cases clearly show that the 2-fold geometries are not only less stable but also independent of the position with respect to the Gr/Ir moiré. This suggests that isolated TCNQ molecules adsorbed atop the Fe-TCNQ layer observed experimentaly are 3-fold coordinated.

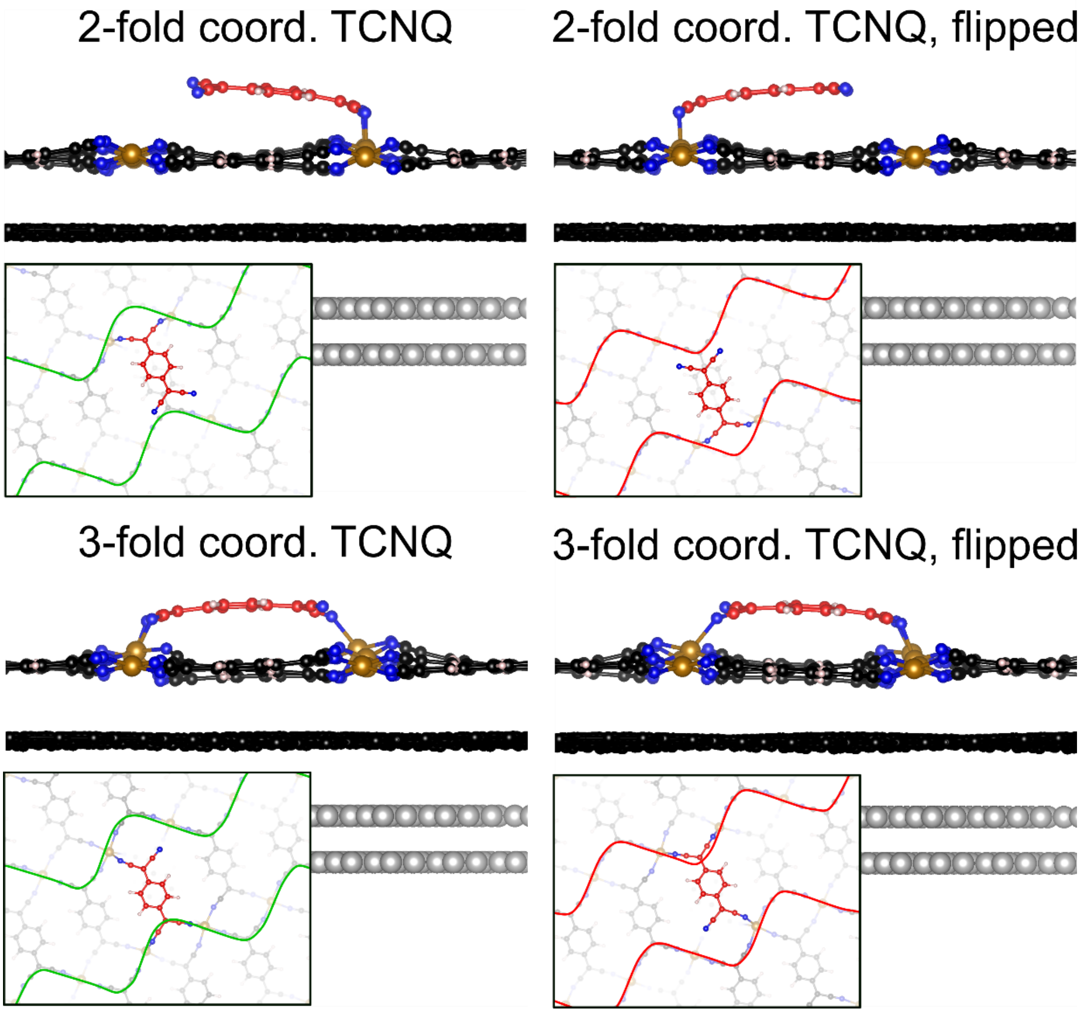

Figure S7: Adsorption geometries tested with DFT calculations: 2-fold coordinated TCNQ molecules (top panels) and 3-fold coordinated TCNQ molecules (bottom panels). Flipped configurations (right panels) are related to the orientation of the zig-zag pattern of the non-planar FeTCNQ layer: red and green wavy lines in insets denote the highest atoms therein.

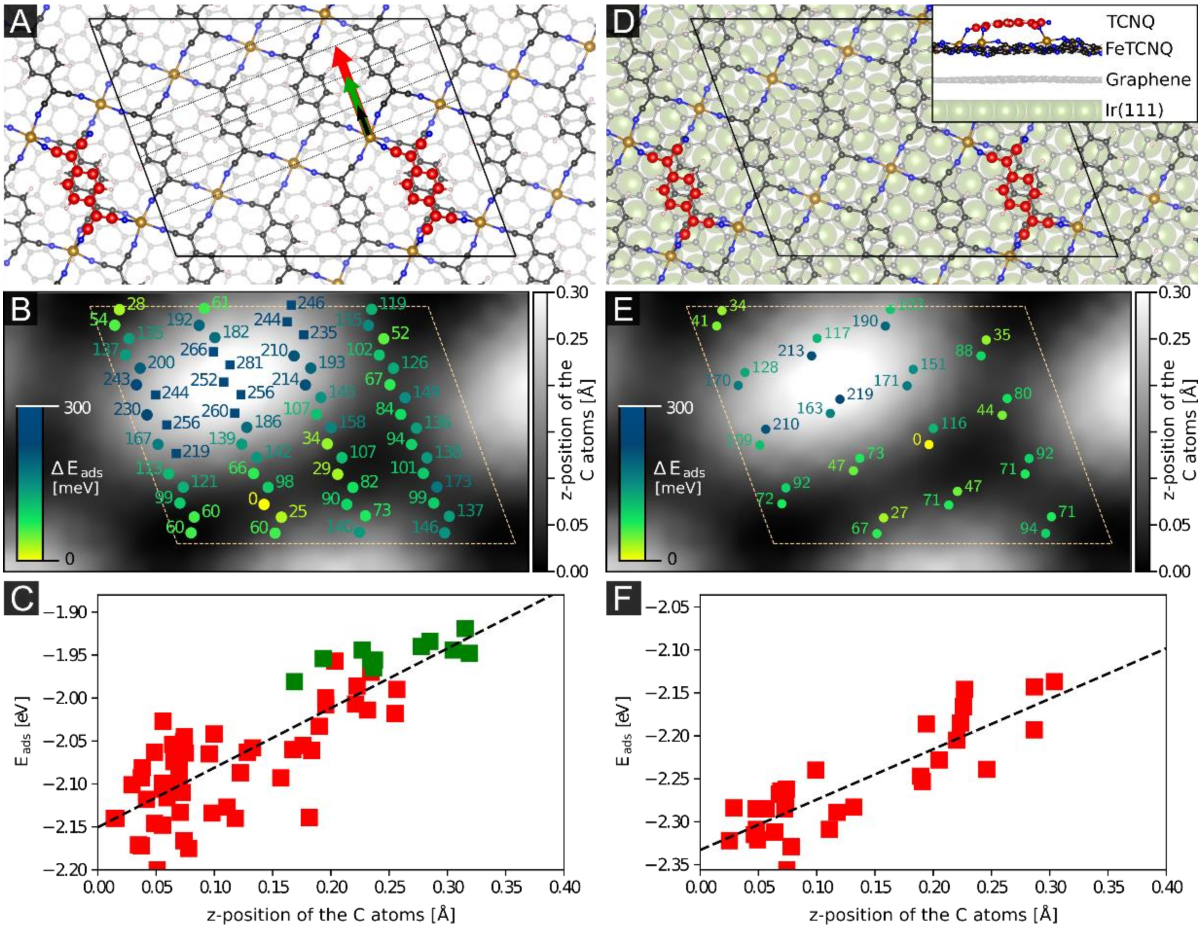

Figure S8: The computational models of TCNQ adsorption atop Fe-TCNQ/Gr (left) and Fe-TCNQ/Gr/Ir (right). (A) The simulated unit cell contains eight Fe_1TCNQ_1 units atop a corrugated Gr moiré unit cell. The adsorbed TCNQ molecule is highlighted in red color. Black, green, and red arrows mark the lateral shifts of the TCNQ/FeTCNQ layer, which were used to generate a denser dataset of adsorption sites with respect to the corrugated graphene. (B) Analysis of the effect of the lateral position of the TCNQ/Fe-TCNQ with respect to the Gr/Ir moiré. The corrugation of the graphene support is highlighted by the gray color coding. The dots and squares represent the center of mass of the adsorbed TCNQ molecule. The numbers indicate the adsorption energy difference of TCNQ/Fe-TCNQ on this with respect to the most stable configuration (in meV). Dots and squares indicate positions where a 3-fold and 2-fold coordination is preferred, respectively. (C) A plot of the TCNQ/Fe-TCNQ adsorption energy vs. the z-position of the underlying graphene. Clearly, the general trend is that the adsorption energy is linearly dependent on the z-position of the underlying graphene. (D-F) The same as in (A-C) but for the computational models that include Ir(111) slab.

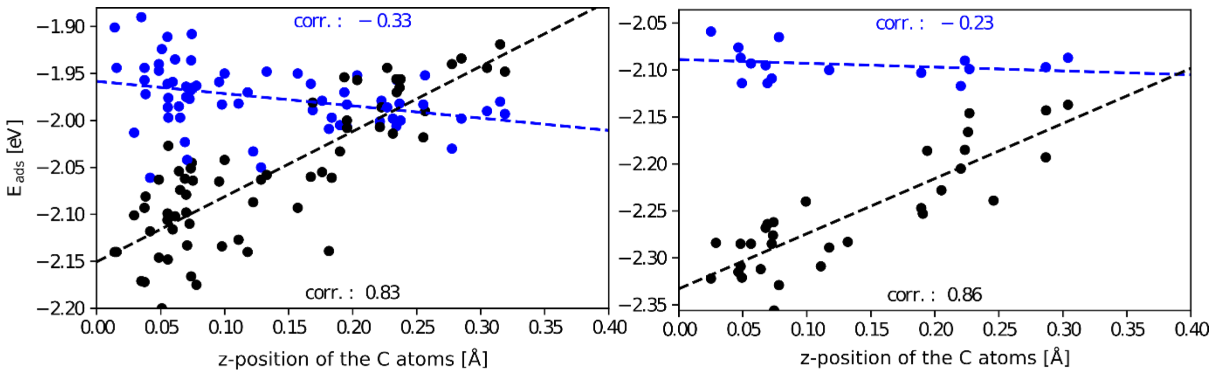

Figure S9: A full dataset of calculated adsorption energies for 2-fold coordinated TCNQ molecules (blue) and 3-fold coordinated TCNQ molecules (black) as the function of a z-position of the underlying graphene. Data in the left (right) panel were obtained from models without (with) the Ir support. Unlike the 2-fold coordinated TCNQ molecules, 3-fold ones show a strong correlation between the adsorption energy and the z-position of the underlying graphene, as shown by trend lines and calculated correlation coefficients.

Supporting Note 7:

Analysis of the electronic structure differences between the models

Figures S10 and Table ST2 indicate that there are no significant differences between the electronic structures of models with the different TCNQ adsorption energies. Figure S10B shows the density of states (DOS) plots of the Fe atoms within two representative models with the TCNQ above the “hill” areas (labeled H1 and H2) and two representative models with the TCNQ above the “valley” areas (labeled V1 and V2), which are shown in Figure S10A. Table ST2 summarizes the Bader charges on the Fe atoms and the adsorbed TCNQ monomers within these four models. In all cases, approximately 1 electron is transferred to the adsorbed TCNQ monomer. The amount of the transferred charge is independent of the TCNQ position with respect to the graphene corrugation. The Bader charge of the Fe atoms involved in the binding to the adsorbed TCNQ monomer (Fe 5-fold) is slightly different from the other Fe atoms within the layer (Fe 4-fold), but this difference is lower than 0.05 e^- in all cases. Figure S11 shows a charge density difference plot for these four models. These charge density difference plots and the Bader analysis clearly indicate that the adsorbed TCNQ accepts charge from the support, as expected from its high electron affinity.

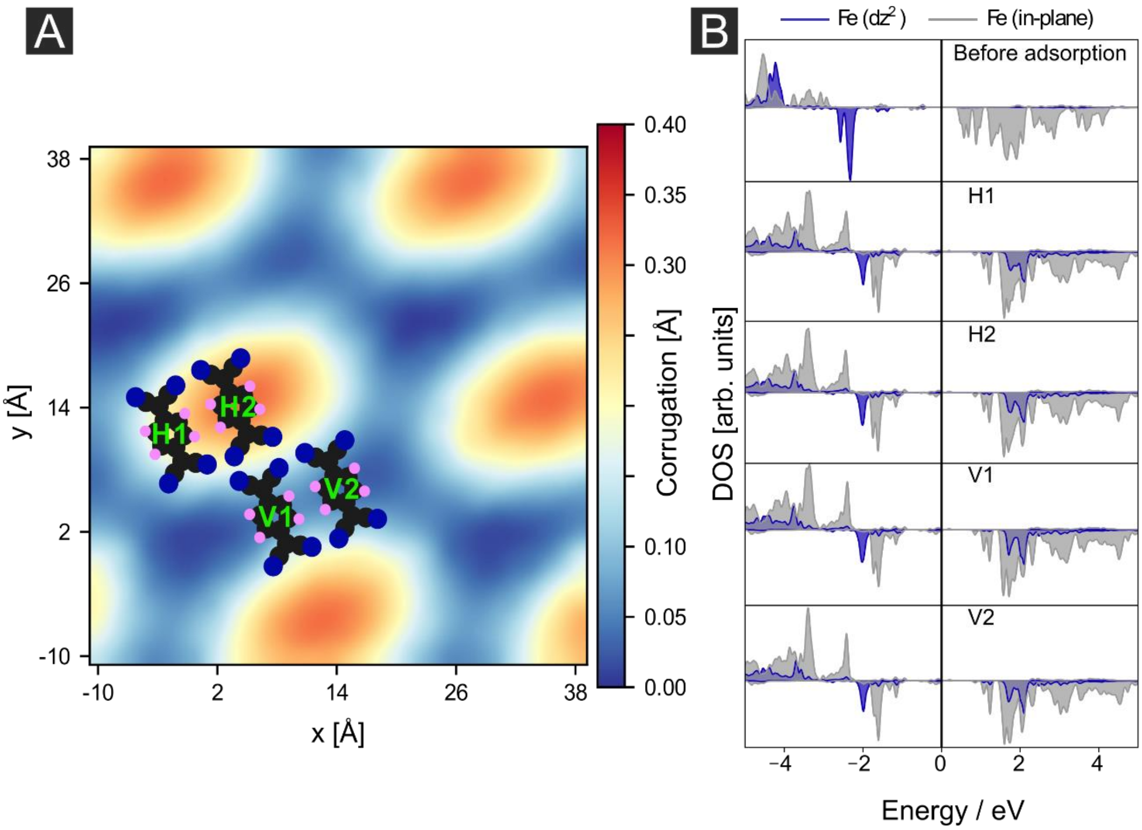

Figure S10: (A) Representative models with the extra TCNQ molecule adsorbed above the ”valley” areas (V1 and V2) and above the ”hill” areas (H1 and H2). Only the position of the extra TCNQ molecule with respect to the graphene corrugation is shown. (B) Projected density of states onto Fe ($3dz^2$) orbitals and the remaining Fe (3d) orbitals for the reference structure before adsorption and for the representative models.

Table ST2: Relative Bader charges (in e^-) for 4-fold and 5-fold Fe atoms in representative models and for the extra TCNQ molecule.

	Fe 4-fold	Fe 5-fold	TCNQ extra
V1	-1.375	-1.421	1.018
V2	-1.376	-1.421	1.018
H1	-1.375	-1.421	1.022
H2	-1.374	-1.422	1.028

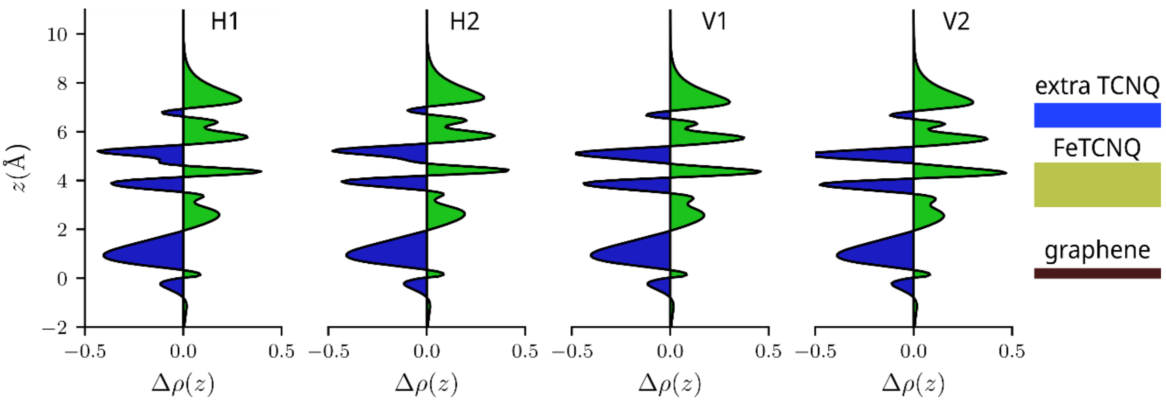

Figure S11: Charge density difference plots for the four representative models shown in Figure S10A. No significant differences were observed between the individual models.

Supporting Note 8:
Additional analysis of the origin of the adsorption energy differences

In the following, we demonstrate that the origin of the adsorption energy differences cannot be described by a simple parameter, but it is rather an effect of delocalized structural differences in combination with dispersion interactions with a graphene substrate. In addition to the calculated Bader charges, projected density of states, and charge density difference plots presented in Supporting Note 7, the Fe-N bond lengths listed in Table ST3 also show no significant differences across the representative models, indicating that changes in the Fe-N bond lengths do not correlate the calculated adsorption energy differences. To better understand the structural differences between the least stable ('H2') and the most stable ('V1') models, we show their structures overlaid in Figure S12. This comparison demonstrates that (i) the corrugation in the FeTCNQ layer follows the graphene corrugation, and (ii) the structural differences are delocalized over the whole layer. Then, in Figure S13, we show the optimal graphene corrugation acquired by the relaxation of the graphene sheet below the TCNQ/FeTCNQ layer. The graphene sheet forms a depression directly below the adsorbed TCNQ monomer, and the associated additional relaxation stabilizes the structure by 0.2 eV with respect to the most stable ('V1') structure. Since the "valley" structures better resemble the optimal structure shown in Figure S13, they are also intuitively expected to be more stable.

Finally, we evaluate how much of the energy difference is due to structural changes and resulting differing ligand field within the TCNQ/FeTCNQ layer and how much is due to the dispersion interaction with the corrugated graphene sheet. The latter, calculated as the difference of adsorption energy of the (fixed) TCNQ/FeTCNQ layer on the corrugated graphene, favors the "V1" structure over the "H2" structure by 106 meV. The effect of structural differences within the TCNQ/FeTCNQ layer can be directly evaluated by comparing the total energies of the TCNQ/FeTCNQ layer of the "V1" and "H2" models, without the graphene layer. This way, the "V1" structure is calculated to be more stable by additional 169 meV than the "H2" structure. Out of this value, only a minor component of 19 meV is attributed to variations in dispersion interactions within the TCNQ+FeTCNQ layer.

To summarize: The total energy difference between the "V1" and "H2" models is 276 meV, out of which 150 meV (54 %) is attributed to structural changes and differing ligand field within the TCNQ/FeTCNQ layer, 106 meV (39 %) is due to differences in the dispersion forces between graphene and TCNQ/FeTCNQ, and the remaining 19 meV (7 %) is due to differences in the dispersion forces within the TCNQ/FeTCNQ.

Table ST3: Average Fe-N bond lengths of the 4-fold and 5-fold Fe atoms in the reference structure without an adsorbed TCNQ monomer and in the representative models depicted in Figure S10A. The last three columns show the bond lengths between the Fe atom and the nitrogen of the adsorbed TCNQ monomer.

	d(Fe-N) 4-fold [Å]	d(Fe-N) 5-fold [Å]	d(Fe-N) extra TCNQ [Å]		
Ref.	2.04	-	-	-	-
H1	2.05	2.13	2.14	2.16	2.19
H2	2.05	2.13	2.14	2.16	2.20
V1	2.05	2.12	2.13	2.14	2.19
V2	2.05	2.12	2.13	2.13	2.19

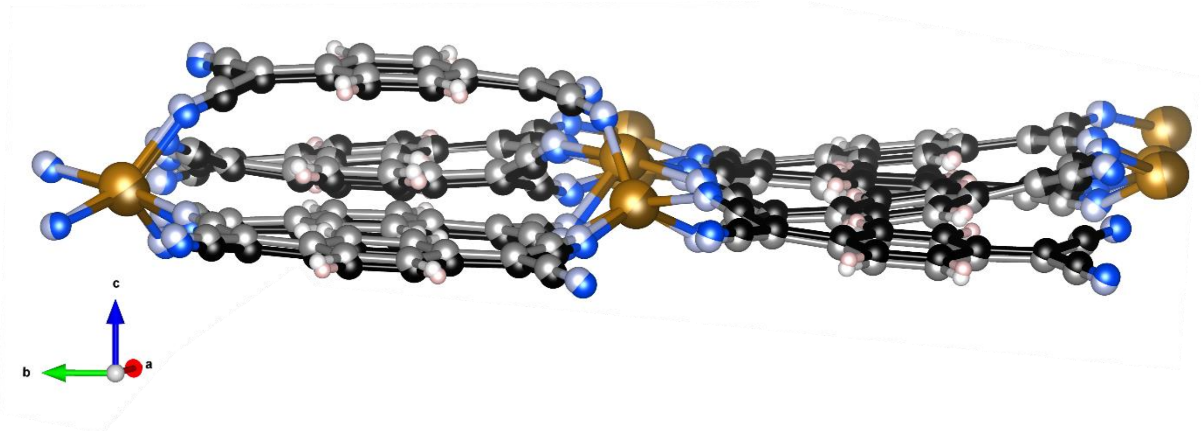

Figure S12: Folded structures of the 'H2' and the 'V1' models rendered in light and dark colors, respectively.

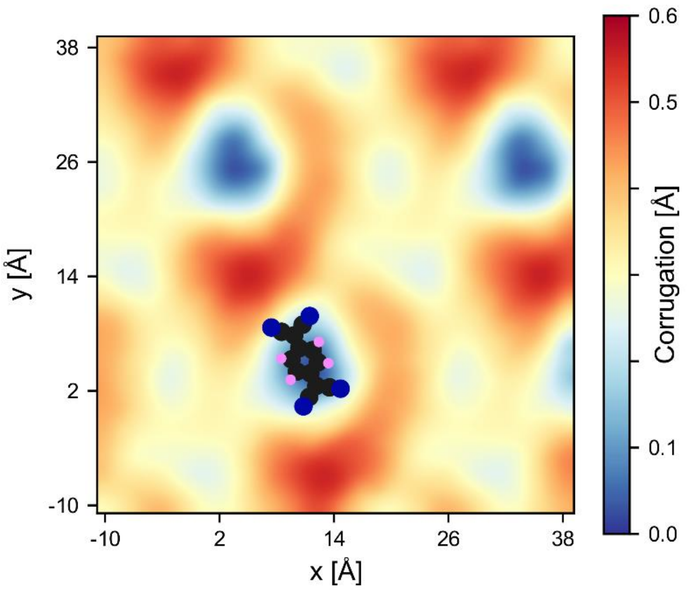

Figure S13: Graphene corrugation imposed by the FeTCNQ layer with the adsorbed TCNQ monomer. The graphene sheet forms a depression below the adsorbed TCNQ molecule.

Supporting Note 9:

Influence of the graphene corrugation on NH_3 adsorption atop Fe-TCNQ

To illustrate that the surprisingly strong effect of support curvature is restricted to large molecules, Figure S14 presents the calculated adsorption energy of ammonia (NH_3) on the Fe cations within the Fe-TCNQ layer on corrugated graphene as a function of the z-position of the underlying graphene. The NH_3 adsorption energy varies by only about 20 meV and does not show any correlation with the support curvature.

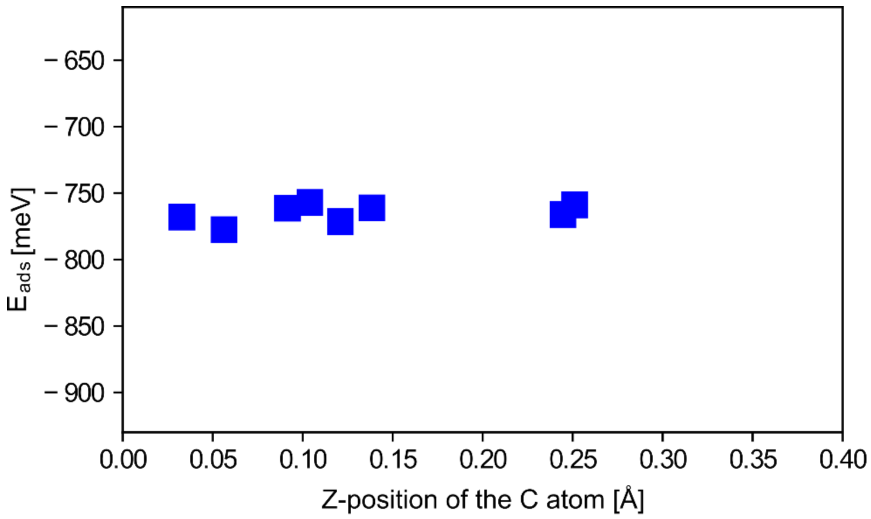

Figure S14: Adsorption energy of the NH_3 molecule adsorbed on the Fe-TCNQ layer vs. the z-position of the underlying graphene.

References

1. Jakub, Z. *et al.* How the Support Defines Properties of 2D Metal–Organic Frameworks: Fe-TCNQ on Graphene versus Au(111). *J Am Chem Soc* **146**, 3471–3482 (2024).
2. Feature Finder, an ImageJ plugin. https://imagej.net/plugins/feature-finder.
3. N'Diaye, A. T., Coraux, J., Plasa, T. N., Busse, C. & Michely, T. Structure of epitaxial graphene on Ir(111). *New J Phys* **10**, 43033 (2008).
